# miRNA-381 regulates renal cancer stem cell properties and sunitinib resistance via targeting SOX4

**DOI:** 10.1016/j.bbrep.2023.101566

**Published:** 2023-11-05

**Authors:** Xiao-jun Lu, Wen-wen Gao, Jia-cheng Li, Sheng-Fei Qin

**Affiliations:** a Department of Urology, Shanghai FourthPeople's Hospital, School of Medicine, Tongji University, Shanghai, 200434, China; b Department of Oncology, Shidong Hospital, Affiliated to University of Shanghai for Science and Technology, Shanghai, China

**Keywords:** Cancer stem cell, Renal cell carcinoma, miR-381, SOX4, Sunitinib

## Abstract

Cancer stem cells (CSCs) are crucial in the pathogenesis of human cancers. Existing studies reported that microRNA (miRNA) modulates the stemness of CSCs. We discovered that renal cell CSCs have suppressed miR-381. Suppression of miR-381 promotes renal cell tumorigenesis and CSC-like properties. Furthermore, the forced expression of miR-381 prevents the renal cell tumorigenesis and CSC-like properties. Mechanistically, renal cell CSCs have been found to interact with SOX4 through miR-381 directly. miR-381 inhibits renal cell CSC-like properties and tumorigenesis via downregulating SOX4. Examination of the patient-derived xenografts (PDX) and patient cohorts reveals that miR-381 may be able to forecast the advantages of Sunitinib in RCC patients. Moreover, the introduction of SOX4 could reverse the sensitivity of miR-381 overexpression RCC cells to Sunitinib-induced cell apoptosis. These results indicated that miR-381 is critical in renal cell CSC-like properties and tumorigenesis, making it the ideal therapeutic target for RCC.

## Highlights

• miR-381 is downregulated in renal CSC-like properties.
• miR-381 inhibits the tumorigenesis and renal CSC-like properties.
• miR-381 inhibits renal CSC-like properties and tumorigenesis via downregulating SOX4.
• miR-381 controls the response to Sunitinib.

## 1 Introduction

One of the most prevalent urinary system tumors, renal cell carcinoma (RCC), responsible for 2%–3% of all cancer cases in adults, is rising by 2 % annually [1]. Approximately 70 % of Renal Cell Carcinoma (RCC) cases are of the clear cell subtype (ccRCC) [2]. Whereas sunitinib (SU)-based chemotherapy is a successful treatment technique for RCC, the 5-year overall survival rate is only 8 % for metastatic and advanced disease because RCC has an inherent tumor resistance [[3], [4], [5], [6]]. In order to enhance the overall prognosis of RCC patients, it is essential to comprehend the SU-resistance mechanism and the particular factors causing SU-resistance RCC.

Tumor-initiating cells (T-ICs), also known as cancer stem cells (CSCs), are a subset of cancer cells that can perpetually renewal themselves and procreate [7,8]. Current research suggests that CSCs control tumor development, growth, metastasis, recurrence, and chemoresistance [[10], [11], [9]]. CSCs are the decisive factors in tumor metastasis and relapse [12,13]. Additionally, earlier research has supported the existence of renal cell CSCs [14]. Nevertheless, the fundamental process that regulates renal cell CSC proliferation remains unknown.

MicroRNAs (miRNAs) are non-coding RNA that regulates downstream targets through post-transcriptional alterations [15]. Numerous investigations have revealed that miRNAs play a role in controlling various biological activities [16]. In certain kinds of cancer, miRNAs act as a tumor suppressor or oncogene [[17], [18], [19]]. It has been reported that miR-381 is involved in multiple solid tumor progression, including RCC [[20], [21], [22]]. The probable function of miR-381 in renal CSCs is still undetermined. The present research found that renal cell CSCs showed suppressed miR-381.

Furthermore, we showed that miR-381, which targets SOX4 *in vitro* and *in vivo*, prevents the tumorigenesis and renal CSC-like properties. More importantly, miR-381 determines the response of Sunitinib in RCC. Thus, the data implicate miR-381 as a Sunitinib medication marker and a therapeutic target for RCC.

## 2 Method

### 2.1 RCC patients' tissues

Participants undergoing the resection of their primary RCC at the Fourth People's Hospital in Shanghai contributed RCC samples. From 2018 to 2020, a total of 30 patients with primary RCC received adjuvant Sunitinib treatment at Shanghai FourthPeople's Hospital. Patient written permission was acquired, and Shanghai Fourth People's Hospital's ethics committee authorized the collection of human samples.

### 2.2 Cell culture

Dulbecco's modified eagle's medium (DMEM) supplemented with 25 μg/mL gentamicin, 2 mM l-glutamine, and 10 % fetal bovine serum (FBS), was utilized for culturing RCC cell lines (HK-2 and CAKI-2) and kept in an incubator at 37 °C with 5 % CO_2_. Ribobio, Shanghai, China's lentiviruses (miR-381 knockdown, miR-381 overexpression, and their control lentiviruses) were used to infect HK-2 and CAKI-2. The stable infectants were then puromycin-screened. Sunitinib (Selleck, S1164), Pazopanib (Selleck, S3012), Sorafenib(Selleck, S7397) was cultured with HK-2 and CAKI-2 cells at a concentration just below their IC50 and raised to 0.2 μM/L per week to determine Sunitinib/Pazopanib/Sorafenib-resistant, Pazopanib-resistant, and Sorafenib-resistant cell lines, respectively. By sustaining three cell lines in an environment of Sunitinib, it was possible to acquire and preserve their resistance to the drug.

### 2.3 RNA interference

Ribobio manufactured siRNAs (small interference RNAs) against SOX4 and NC (negative control) siRNA (Ribobio, Shanghai, China). SOX4 siRNA target sequences are 5′-GGACAGACGAAGAGUUUAATT-3''. Utilizing a siRNA transfection reagent and following the supplier's guidelines, at a final concentration of 200 nM, the siRNAs were transfected into the RCC cells. WB confirmed the validity of gene suppression. Suppression of miR-381 and their regulation After SOX4 siRNA or negative control were transfected into RCC cells, spheroids formed, and an *in vitro* and *in vivo* LDA was performed.

### 2.4 *In vivo* experiments

Female NOD-SCID mice (4 weeks old) were maintained and fed under standard pathogen-free circumstances by Wuhan servicebio Co. in China. RCC cells were successively diluted to the relevant doses—1x10 [3], 5 × 10^3^, 1 × 10^4^, and 5x10^4^—for the *in vivo* limited dilution assay and then mixed with 100 μl of Matrigel gel 1:1 ratio. After that, NOD-SCID mice (n = 5, randomly assigned) received subcutaneous injections of the mixed cells. The mice were slaughtered after 2 months, and the tumors were measured. All animal procedures complied with standards for the National Institutes of Health guide for the care and use of Laboratory animals (NIH Publications No. 8023, revised 1978), and the Shanghai FourthPeople's Hospital Ethics Committee approved the methods.

### 2.5 PDX model

After monitoring the development of xenografted tumors, the female mice (4 weeks old) were euthanized 10 weeks after the injection. As reported earlier, primary tumor samples were collected for the PDX model (patient-derived xenograft) for xenograft development [23]. The xenografts (150–200 mm^3^) were then size-matched and randomly assigned to one of two treatment groups (Sunitinib or Vehicle). For 24 days, the mice were given intraperitoneal injections of sunitinib (4 mg/kg) or vehicle. The tumor volume was measured with a caliper twice weekly using the Volume = = π/6*L*W2 formula (where; L = longest and W = shortest tumor axis). The mice were subjected to humanely death by CO_2_ after the PDX volume reached roughly 1500 mm^3^, and the tumors were cut into pieces or frozen for later study. All animal procedures complied with standards for the National Institutes of Health guide for the care and use of Laboratory animals (NIH Publications No. 8023, revised 1978), and the Shanghai FourthPeople's Hospital Ethics Committee approved the methods.

### 2.6 Spheroid formation assay

For one week, the RCC cells were cultured in DMEM/F12 (Gibco) enriched with 20 ng/mL EGF, 20 ng/mL bFGF, and 1 % FBS. The RCC cells were plated on 96-well culture plates (Corning Incorporated Life Sciences, USA) at 300 cells per well density. Spheroids were counted, and illustrative images were displayed. A total of three times, these findings were replicated.

### 2.7 Limiting dilution assay *in vitro* (LDA)

The RCC cells were cultured in DMEM/F12 (Gibco) treated with 20 ng/mL EGF, 20 ng/mL bFGF, and 1 % FBS for 1 week after being plated on 96-well culture plates with 2, 4, 8, 16, 32, and 64 cells/well (n = 8). ELDA program (http://bioinf.wehi.edu.au/software/elda/index.html) was used to calculate the percentage of CSCs [27]. The results were obtained in triplicate.

### 2.8 Apoptosis assay

Sunitinib (10 μM) was administered to RCC cells with miR-381 upregulation and their control for 48 h. Next, Annexin V and 7-AAD staining was performed for 15 min at room temperature in the dark. Following the company's directions, a flow cytometer and Annexin VFITC Apoptosis Detection Kit I (BD Pharmingen, San Diego, CA) were employed for apoptotic cell identification.

### 2.9 Luciferase reporter assay

The conserved miR-381-binding sites from the SOX43′UTR were introduced into a luciferase reporter plasmid in a 500-bp segment. Changes were made to the putative miR-381-binding base sequence “UGCUGGA” to “GGGGGGA” in the SOX43′UTR mutant luciferase plasmid. NEXT, the SOX4 mutant 3′UTR fragment's 500-bp segment was introduced into a luciferase reporter plasmid. RCC cells with miR-381 knockdown and their control cells were planted in 24-well plates for the luciferase reporter experiment. Following the company's guidelines, they were co-transfected using Lipofectamine 2000 (Invitrogen) with 2 ng per well of the pRLCMV vector, 2 ng per well of pRLCMV vector (internal control, Promega), 20 ng per well of miR-381 precursor molecules or control precursor (Applied Biosystems), and 100 ng per well of the resulting luciferase UTR-report vector. The Dual-Luciferase Assay Reporter System (Promega) was used to measure the relative luciferase activity after the cells had been lysed for 24 h.

### 2.10 Real-time PCR (RT-PCR) assay

TRIZOL (Invitrogen) was utilized to extract total RNA from cells or tissues per the company's directions. A NanoDrop ND-1000 UV spectrophotometer was used to evaluate the purity of the RNA, and agarose gel electrophoresis was used to confirm the integrity of the RNA. The Promega M-MLV RTase cDNA Synthesis Kit performed reverse transcription on the isolated RNA to produce cDNA. RT-PCR analysis was conducted using a LightCycler 480 System and an SYBR Green PCR Kit from Roche. Denaturation stage PCR settings included 1 cycle for 5s at 95 °C, followed by 40 cycles for 15s at 95 °C, annealing for 30s at 60 °C, and extension for 30s at 72 °C. Melting curves obtained after the experiment validated the specificity of the primers (as given below). Each sample was measured three times in biological duplicates.

U6

Forward: 5″ ATTGGAACGATACAGAGAAGATT 3''.

SOX4

Forward: 5″ GCACTAGGACGTCTGCCTTT 3″,

reverse: 5″ ACACGGCATATTGCACAGGA 3''.

β-actin

Forward: 5″ GGCCCAGAATGCAGTTCGCCTT 3″,

reverse: 5″ AATGGCACCCTGCTCACGCA 3''.

### 2.11 Western blotting (WB) assay

Specimens were collected using cell lysis buffer and discarded as previously reported [23]. Sodium dodecyl sulfate-polyacrylamide gel electrophoresis (SDS-PAGE) separated 25 μg proteins, which were transferred to the nitrocellulose (NC) membrane. Following blocking with 5 % nonfat milk, the NC membrane was incubated with the primary antibody, followed by the IRDye 800CW-conjugated secondary antibody. The fluorescein intensity was detected using the LI-COR imaging system (LI-COR Biosciences). The primary antibodies were SOX4 (1:1000, ab86809, Abcam), PARP (1:1000; 13371-1-AP, Proteintech), β-actin (1:1000; 27309-1-AP, Proteintech).

### 2.12 Flow-cytometric analysis

For CD133 and CD90 positive cells sorting, primary RCC patients' cells were incubated with the primary anti-CD133 (Biolegend, Inc., San Diego, CA) or anti-CD90 (Biolegend, Inc., San Diego, CA) for 30 min at room temperature. The cells were then subjected to flow cytometry using a MoFlo XDP cell sorter from Beckman Coulter (Indianapolis, IN, USA) according to the manufacturer's instructions. The sorted cells from three independent experiments were subjected to Real-time PCR assay. The results were repeated for three times.

### 2.13 Data analysis

At least three different runs of each experiment were completed. Data were shown as the mean ± standard deviation. For all statistical calculations, GraphPad Prism software was utilized. A *t*-test or the Bonferroni Multiple Comparisons Test was used for statistical analysis. The statistical significance threshold has been defined as a p-value less than or equal to 0.05.

## 3 Result

### 3.1 miR-381 is downregulated in renal CSC-like properties

**Fig. 1:**
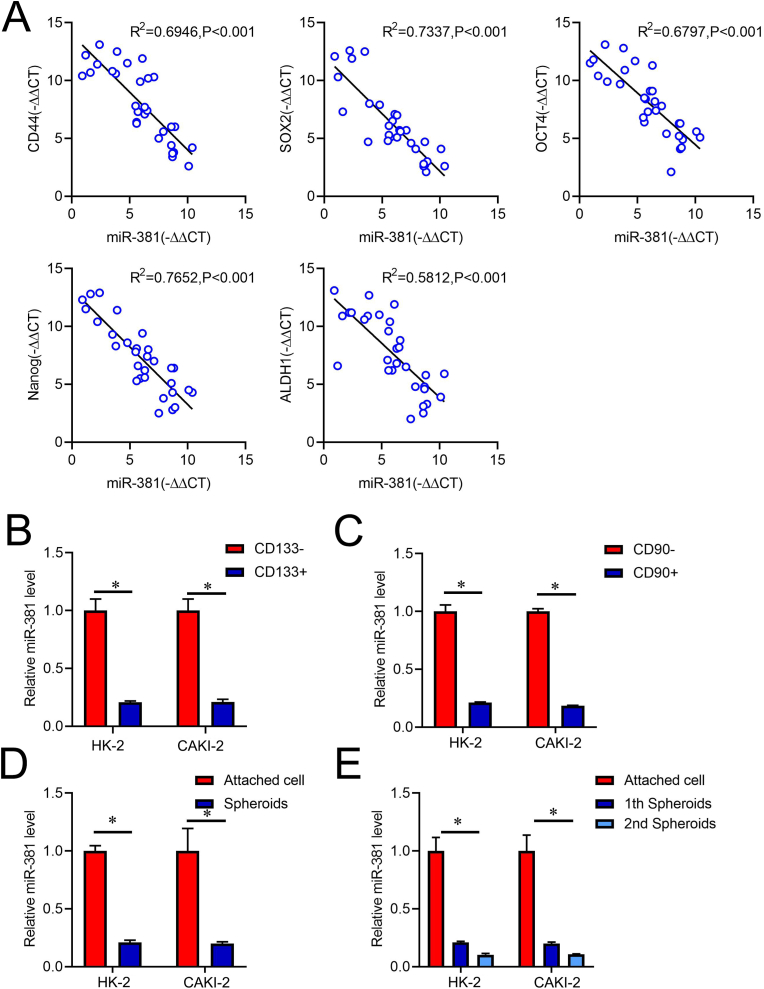
**miR-381 expression is downregulated in renal CSC-like properties**. **A**. RT-PCR was used to examine the relationship between miR-381 and *CD44, Sox2, Oct4, Nanog,* and *ALDH1* levels in primary RCC tissues (n = 30). The measurements were adjusted to β-actin as △Ct and Spearman's correlation was used to assess them. **B**. miR-381 expression in CD133^+^ RCC cells and CD133^-^ RCC cells were analyzed by real-time PCR assay. **C**. miR-381 expression in CD90^+^ RCC cells and CD90^−^ RCC cells were compared by real-time PCR assay. **D** and **E**. RT-PCR determined miR-381 expression in primary RCC spheroids and adherent cells.

As shown in Fig. 1A, miR-381 levels have been demonstrated to be inversely linked with the expression of defined cancer stem cell marker genes [24], including *CD44, Sox2, Oct4, Nanog,* and *ALDH1* in RCC tissues. Existing evidence showed that CD133 and CD90 are well-known CSCs markers [24,25], we enriched CD133^+^ and CD90^+^ renal Cell Carcinoma cells via flow cytometry sorting. As expected, CD133^+^ or CD90^+^ RCC cells displayed significantly high *CD44, Sox2, Oct4, Nanog, and ALDH1* expresion than CD133^-^ or CD90^−^ RCC cells, indcating that CD133^+^ or CD90^+^ RCC cells existed Renal cell CSCs characteristic(Figs. S1A and B). As shown in Fig. 1B&C, miR-381 levels were significantly downregulated in isolated CD133^+^ or CD90^+^ RCC cells compared with CD133^-^ or CD90^−^RCC cells. Following, as shown in Fig. S1C&D, Pearson correlation analysis revealed that miR-381 levels were negatively correlated with the expression of CD133 and CD90 in primary RCC tissues. Meanwhile, sphere formation is a selection method that enriches CSCs [5], miR-381 expression was dramatically reduced in RCC spheres than in the adherent RCC cells (Fig. 1D). Notably, miR-381 expression was decreased gradually in RCC spheroids' sequential expansions (Fig. 1E).

### 3.2 miR-381 inhibits the tumorigenesis and renal CSC-like properties

**Fig. 2:**
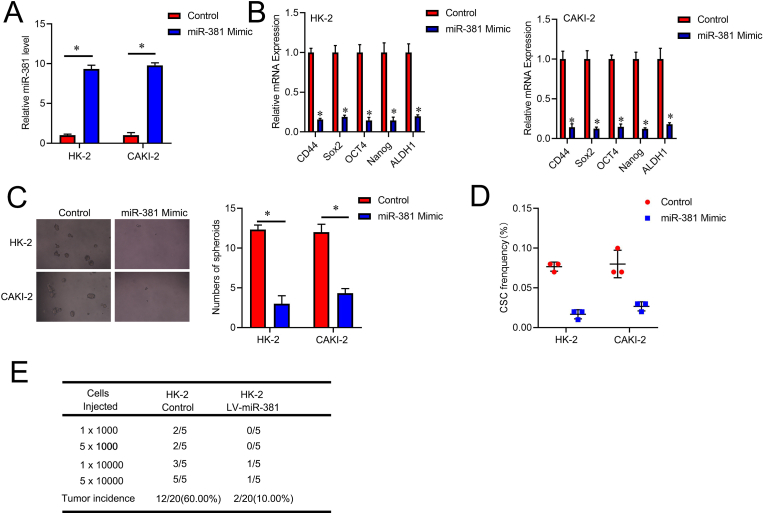
**miR-381 upregulation suppresses the tumorigenesis and renal CSC-like properties. A**. The miR-381 overexpression virus was used to infect HK-2 and CAKI-2 cells, and RT-PCR was used to assess the overexpression effect. **B**. Analyses of the *CD44, Sox2, Oct4, Nanog*, and *ALDH1* mRNA expression in RCC cells with and without upregulation of miR-381. **C**. Spheroids formation assay of miR-381 overexpression cells and control RCC cells. Representative images of spheres are shown, scale bar = 50 μM. **D**. The LDA examined the fraction of renal cell CSCs in control RCC cells and miR-381 upregulated cells. **E**. miR-381 overexpression and control RCC cells were processed for an *in vivo* limited dilution experiment. Tumors were tracked for two months (n = 5).

HK-2 and CAKI-2 cells were infected with the miR-381 overexpression virus, and the RT-PCR method was used to determine the expected molecular role of miR-381 in renal cell CSCs (Fig. 2A). Compared with the control RCC cells, miR-381 overexpression cells showed decreased defined cancer stem cell-associated marker genes (Fig. 2B). miR-381 overexpression cells exhibited impaired sphere-formation rate (Fig. 2C). An *in vitro* LDA revealed that the quantity of CSCs in miR-381 overexpression RCC cells was significantly decreased (Fig. 2D). Notably, *in vivo,* LDA showed that the tumorigenesis capacity was dramatically impaired by miR-381 overexpression in renal cancer cells (Fig. 2E).

**Fig. 3:**
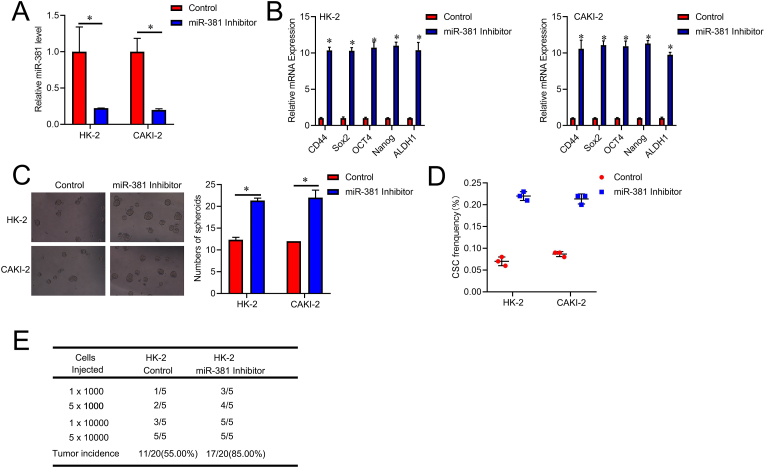
**miR-381 knockdown promotes the tumorigenesis and renal CSC-like properties. A**. HK-2 and CAKI-2 cells were infected with miR-381 knockdown virus and were assessed through RT-PCR assay. **B**. The *CD44, Sox2, Oct4, Nanog*, and *ALDH1* mRNA expression levels were examined for miR-381 suppression and control RCC cells. **C**. Spheroids formation assay of miR-381 knockdown cells and control RCC cells. Representative images of spheres are shown, scale bar = 50 μM. **D**. An *in vitro* LDA assessed the proportion of renal cell CSCs for miR-381 suppression cells versus control RCC cells. **E**. LDA on miR-381 knockdown cells and control RCC cells *in vivo*.

Next, the miR-381 suppression virus infects HK-2 and CAKI-2 cells. The interference impact was then assessed using an RT-PCR experiment (Fig. 3A). MiR-381 suppression cells displayed elevated defined cancer stem cell-associated marker genes compared to the control RCC cells (Fig. 3B). Moreover, miR-381 knockdown cells exhibited enhanced sphere-formation rate (Fig. 3C). The fraction of CSCs was much higher in miR-381 suppression RCC cells, according to an *in vitro* LDA (Fig. 3D). In addition, an *in vivo* LDA experiment showed that miR-381 suppression in renal cancer cells significantly increased the ability for tumorigenesis (Fig. 3E). These findings demonstrated that miR-381 inhibits renal CSC-like properties.

### 3.3 MiR-381 targets the 3′-UTR of SOX4 to reduce its expression

**Fig. 4:**
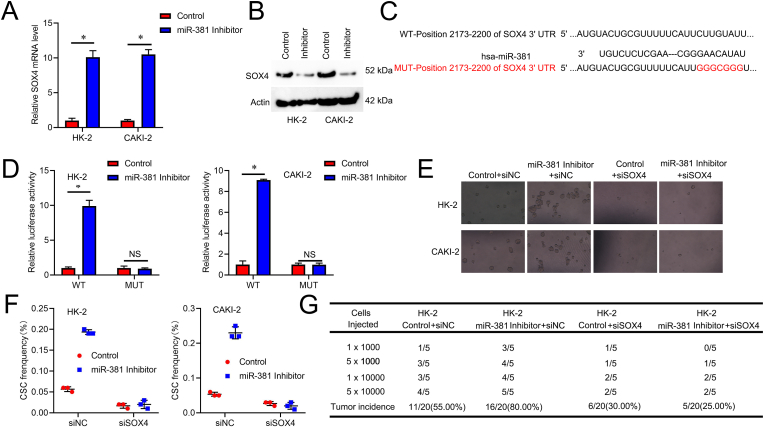
**SOX4 is required for miR-381-mediated the tumorigenesis and renal CSC-like properties. A**. RT-PCR analysis of SOX4 in control RCC and miR-381 knockdown cells. **B**. Western blot analysis of SOX4 in miR-381 suppression cells and control RCC cells. **C**. Prediction of miR-381 binding sites in the 3′-UTR of the SOX4 gene and the nucleotides altered in the SOX4-3′UTR mutant using Target Scan. **D**. In miR-381 suppression cells and control RCC cells, the luciferase activity of the SOX4-WT or SOX4-Mut 3′-UTR was evaluated, and the relative activity was displayed. **E**. Spheroids were formed after miR-381 knockdown cells, and control RCC cells were transfected with SOX4 siRNA or negative control, scale bar = 50 μM. **F**. *In vitro,* assays were performed on miR-381 knockdown cells and controlled RCC cells treated with SOX4 siRNA or negative control. **G**. The LDAs were performed on miR-381 knockdown cells and control RCC cells treated with SOX4 siRNA or negative control.

Afterward, we used TargetScan to estimate probable direct targets of miR-381. We discovered numerous genes with potential miR-381 binding sites, and one of these genes, SOX4, was thought to regulate CSCs [26]. Analysis of RT-PCR and western blots revealed that miR-381 suppression raised the protein levels and mRNA of SOX4 (Fig. 4A and B). The full-length 3′-untranslated region (3′-UTR) of the SOX4 gene was cloned into the area downstream of the Renilla luciferase gene to validate that SOX4 is a direct target of miR-381 (Fig. 4C). When the reporter constructs carried the wild-type 3′-UTR, the miR-381 knockdown increased the luciferase activity(Fig. 4D). Nevertheless, miR-381-mediated increase in luciferase activity was eliminated by mutation of the miR-381 targets region.

### 3.4 miR-381 inhibits renal CSC-like properties and tumorigenesis via targeting SOX4

The unique SOX4 siRNA was further transfected to miR-381 suppression and control RCC cells to validate the functional relationship between miR-381 and SOX4. As anticipated, a specific SOX4 siRNA reduced the sphere-formation rate difference between RCC cells with miR-381 suppression and control cells (Fig. 4E). A particular SOX4 siRNA also eliminated the CSC proportion difference between RCC cells with miR-381 suppression and control cells (Fig. 4F). Furthermore, an unusual SOX4 siRNA eliminated the tumorigenesis potential difference between miR-381 suppression and control RCC cells (Fig. 4G). The results presented here showed that SOX4 was necessary for miR-381-mediated renal cell CSC growth.

### 3.5 miR-381 controls the response to sunitinib In RCC cells

**Fig. 5:**
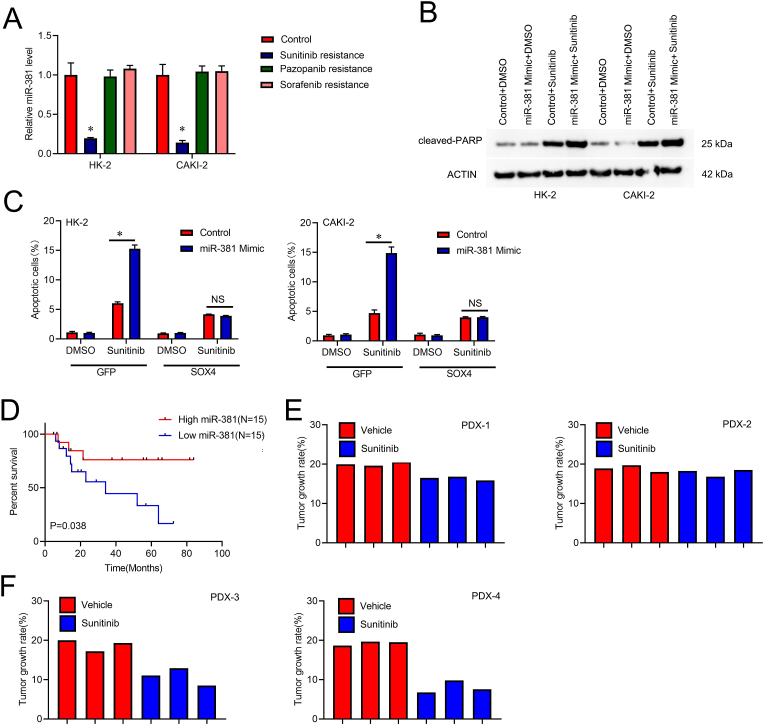
**Sunitinib sensitivity in RCC cells is determined by miR-381. A**. RT-PCR assay of miR-381 Sunitinib/Pazopanib/Sorafenib-resistant cells and control RCC cells. **B**. Sunitinib (1.0 μM/L) was applied to miR-381 overexpression and control RCC cells for 48 h before the Western blot experiment. **C**. The control RCC and MiR-381 imitated cells were infected with SOX4 upregulation or a negative control virus, then treated for 48 h with Sunitinib (1.0 μM/L). The fraction of apoptotic cells was determined using the FC method. **D**. Kaplan-Meier analysis was employed to compare overall survival between miR-381-high (n = 15) and miR-381-low (n = 15) groups. **E**. Sunitinib or saline was given to PDXs generated from primary RCCs with low miR-381 levels (n = 3 in each group; 24 days). The xenograft growth rate has been determined. **F**. Sunitinib or saline was given to PDXs generated from primary RCCs with high miR-381 levels (n = 3 in each group; 24 days). The rate The xenograft growth rate was determined.

Accumulating evidence suggests that CSCs have a role in the control of chemoresistance [27]. We further explore the relationship between miR-381 and chemoresistance in RCC. Our previous construction of Sunitinib-resistant, Pazopanib-resistant, and Sorafenib-resistant cell lines were performed and the miR-381 levels were significantly decreased in Sunitinib-resistant RCC cell lines, while the miR-381 levels were no significant changed in Pazopanib-resistant,and sorafenib-resistant cell lines (Fig. 5A). When subjected to Sunitinib, miR-381 upregulation RCC cells showed a significantly higher protein level of cleaved-PARP than control RCC cells (Fig. 5B). Additionally, SOX4 might reduce the sensitivity of RCC cells exhibiting miR-381 overexpression to sunitinib-induced cell death (Fig. 5C). Importantly, we discovered that individuals with RCC showing elevated miR-381 expression demonstrated prolonged survival after Sunitinib therapy. We employed Kaplan-Meier analysis of RCC patients who received the treatment after surgery (Fig. 5D). Furthermore, the patient-derive xenograft (PDX) model showed that miR-381 low RCC tissues resisted Sunitinib treatment (Fig. 5E). In contrast, miR-381 high RCC tissues were sensitive to Sunitinib treatment (Fig. 5F). Overall, the findings suggested that miR-381 might be a trustworthy indicator of the effectiveness of sunitinib therapy.

## 4 Discussion

In the present investigation, we discovered that primary renal cell CSCs have downregulated miR-381 expression. MiR-381 is crucial for the tumorigenicity and CSC-like properties of renal cells. Further inquiry into the process showed a unique miR-381/SOX4 regulation mechanism in renal CSCs. MiR-381 plays a significant role in determining how RCC cells react to Sunitinib therapy, and miR-381 upregulation eliminated Sunitinib sensitivity. Further research into the patient cohort revealed that miR-381 might predict the therapeutic effectiveness of Sunitinib in RCC individuals.

According to mounting data, miRNAs have recently been found to have important functions in controlling T-ICs or CSCs. Both loss and gain of miRNA functions contribute to CSCs or T-ICs self-renewal and tumorigenesis [[28], [29], [30], [31]]. MiR-206, for example, decreases EGFR expression to limit the formation of liver cancer stem cells [32]. MiR-186 inhibits the development of liver cancer stem cells by controlling the expression of PTPN11 [30]. Through controlling CD24, miR-146a/AKT/β-Catenin activation controls the cancer stem cell phenotype in oral squamous cell carcinoma [33]. Our findings showed that miR-381 expression decreased in renal cell CSCs in the current research. Forced miR-381 expression decreased stemness associated with transcription factors and CSCs markers. According to our findings, the overexpression of miR-381 hampered the tumorigenicity and CSC-like properties of renal cells. The present research outcomes suggest that miR-381 is essential in the growth of renal cell CSCs, implying that miR-381 is a practical treatment option.

We initiated this investigation by using bioinformatics to identify miR-381-targeted genes. We also discovered that miR-381 might suppress SOX4 expression in renal cell CSCs for several potential genes. SOX4 has been demonstrated to be dysregulated in several malignancies [34]. Recent studies showed an important role for SOX4 in epithelial–mesenchymal transition (EMT) [35]. Furthermore, SOX4 has been suggested to be implicated in the modulation of CSCs or T-ICs [30]. In the present research, we discovered that miR-381 inhibits SOX4 expression via binding to its 3′UTR in renal cell CSCs. Our findings show that SOX4 is a direct target gene of miR-381 for the first time. Rescue experiments revealed that miR-381 restricted the development of renal cell CSCs by, at least in part, suppressing SOX4 expression. Sunitinib has been recommended to treat mccRCC over 10 years [36]. However, several late-stage RCC patients acquire resistance to Sunitinib very quickly after starting treatment. As a result, investigating the genetic cause of Sunitinib resistance and identifying reliable indicators of Sunitinib responsiveness is critical. We discovered that RCC cells with miR-381 overexpression are more vulnerable to sunitinib-induced proliferation suppression and mortality. In addition, a Kaplan-Meier analysis of RCC individuals who received Sunitinib therapy following surgery revealed that RCC individuals exhibiting elevated miR-381 expression showed a longer survival time after Sunitinib administration. Moreover, the PDX model also showed that miR-381 high RCC patients benefited from Sunitinib administration. As a result, before selecting a course of therapy, it is prudent to assess miR-381 expression in RCC tumors to determine individuals that could benefit from Sunitinib therapy, which needs additional exploration in biomarker-guided clinical studies.

## Funding

None.

## Availability of data and materials

The data in the current study are available from the corresponding authors upon reasonable request.

## Declaration of competing interest

The authors have no conflict of interest.

## Data Availability

The data that has been used is confidential.
