# Supplementary material for: miRNA-381 regulates renal cancer stem cell properties and sunitinib resistance via targeting SOX4
Source: Biochem Biophys Rep. 2023 Nov 5;36:101566. doi: 10.1016/j.bbrep.2023.101566 (PMC10641571; doi:10.1016/j.bbrep.2023.101566)
Supplement: Multimedia component 1 [file mmc1.docx]

**miRNA-381 Regulates Renal Cancer Stem Cell Properties and Sunitinib resistance via Targeting SOX4**

Xiao-jun Lu1, Wen-wen Gao2, Jia-cheng Li1, Sheng-Fei Qin1

1. Department of Urology, Shanghai FourthPeople's Hospital, School of Medicine, Tongji University, Shanghai 200434, China

2. Department of Oncology, Shidong hospital, Affiliated to University of Shanghai for Science and Technology, Shanghai, China

Correspondence: Sheng-Fei Qin, Department of Urology, Shanghai FourthPeople's Hospital, School of Medicine, Tongji University, Shanghai 200434, China email: qinsf2020@163.com

**
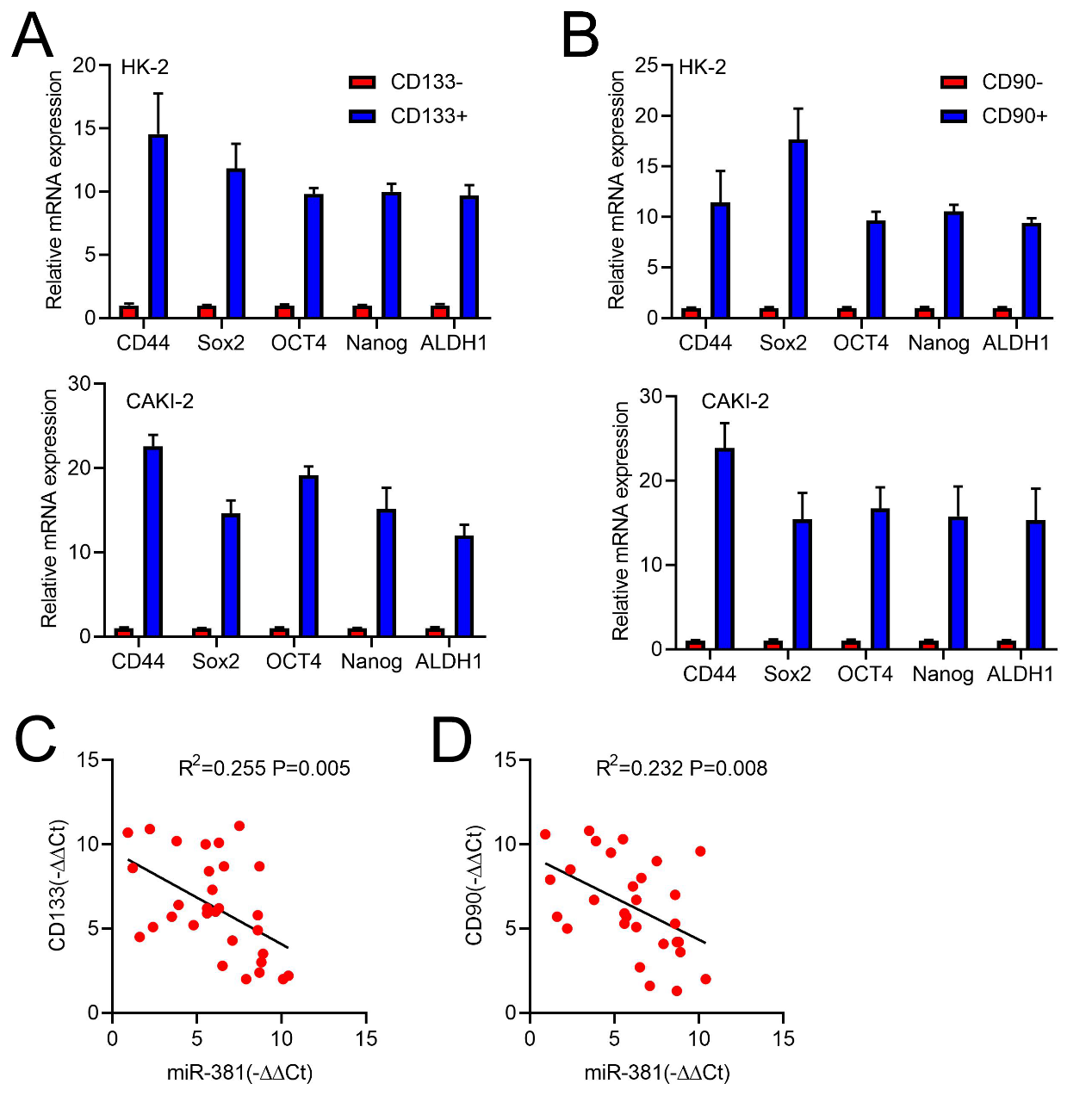
Supplement Figure 1**

A. *CD44, Sox2, Oct4, Nanog,* and *ALDH1* expression in CD133^+^ RCC cells and CD133^-^ RCC cells were analyzed by real-time PCR assay. B*. CD44, Sox2, Oct4, Nanog,* and *ALDH1* expression in CD133^+^ RCC cells and CD133^-^ RCC cells were analyzed by real-time PCR assay. C. The correlation between the level of miR-381 and CD90 in primary RCC tissues was determined by real-time PCR analysis. Data were normalized to β-actin as △Ct and analyzed by Spearman’s correlation analysis. D. miR-381 expression in CD133+ RCC cells and CD133- RCC tissues were analyzed by real-time PCR assay.
